# Long-term effectiveness and overdiagnosis in the NHS breast screening programme: a cohort study with up to 33 years of follow-up

**DOI:** 10.1038/s41416-026-03493-z

**Published:** 2026-06-26

**Authors:** Amanda Dibden, Dharmishta Parmar, Rhian Gabe, Robert A. Smith, Sue M. Moss, Louise E. Johns, Stephen W. Duffy

**Affiliations:** 1 Centre for Cancer Screening, Prevention and Early Diagnosis, Wolfson Institute of Population Health, Queen Mary University of London, London, UK; 2 Center for Early Cancer Detection, American Cancer Society, Atlanta, GA USA; 3 Division of Genetics and Epidemiology, The Institute of Cancer Research, Sutton, UK

**Keywords:** Cancer screening, Cancer prevention, Breast cancer, Cancer epidemiology

## Abstract

**Background:**

Population-based mammographic screening programmes have been operating for over 30 years and whilst many studies have assessed the short- to medium-term effectiveness of screening, few have assessed their long-term effectiveness. The aim of this study was to assess the long-term effectiveness of the NHS Breast Screening Programme in England and Wales.

**Methods:**

2,509,384 women aged 49–64 years and uninvited to screening upon study entry were studied, with screening status changing on invitation and attendance. Women were followed for breast cancer diagnosis and death for a maximum of 33 years. Poisson regression was used to assess the effect of screening on breast cancer incidence and mortality compared to an uninvited comparison group, and overdiagnosis.

**Results:**

After adjustment for confounders, invitation to screening was associated with a 28% reduction in breast cancer mortality (RR: 0.72; 95% CI: 0.71–0.74) and attendance at screening was associated with a 33% reduction (RR: 0.67; 95% CI: 0.65–0.69). There was a 4% excess of breast cancer diagnoses associated with being screened (RR: 1.04; 95% CI: 1.02–1.07) with at least 5 years follow-up post-screening.

**Conclusion:**

These findings indicate that screening continues to be effective in reducing breast cancer mortality many years after cessation of screening and that the level of overdiagnosis associated with long-term follow-up is low.

## Introduction

Population-based mammographic screening programmes were introduced in many countries following early results from randomised controlled trials (RCTs) which showed that invitation to screening reduced breast cancer mortality by 20– 30% [1, 2]. Subsequent studies with longer follow-up have demonstrated that whilst the relative benefit of screening remained stable, the absolute number of breast cancer deaths prevented increased with increasing length of follow-up [3]. This is because breast cancer progression can be slow, and thus screening not only prevents deaths in the immediate period, but 10–25 years post diagnosis.

Observational studies have shown that the effectiveness of population-based screening programmes is in keeping with the results of the early RCTs [4–7]. A recent review of worldwide incidence-based mortality (IBM) studies estimated that invitation to screening is associated with a 22% reduction in breast cancer mortality and a corresponding 33% reduction with attendance at screening [4]. It should be noted, however, that effects varied considerably among studies, with some finding little or no reduction in breast cancer mortality with invitation to screening, and others very large effects, of the order of 40–50% reductions.

In the UK, the NHS Breast Screening Programme (NHSBSP) was introduced in 1988. The NHSBSP differs from European and global counterparts as women are invited every three years rather than biennially [8]. Studies assessing the effectiveness of the UK programme reported an estimated reduction in breast cancer mortality following invitation to screening of 21–27% [9–11] and in those who attend, 30–42% [12–15]. The majority of these studies, however, were case-control studies in which only attendance to screening was measured. It can often be useful to measure the policy effect of offering screening to the whole population (effect of invitation to screening) [16]. Cohort studies offer the opportunity to estimate the effect of both invitation to and attendance at screening, but require long-term follow-up to achieve adequate statistical power and to measure the maximum benefit of attendance to screening.

To date, only two cohort studies have sought to assess the effectiveness of the UK NHSBSP [9, 11]. The first was a non-randomised study of effect of mammographic screening and education regarding breast self-examination at eight screening centres in England and Scotland, in women followed for up to 16 years [11]. The study was established in 1979, before the introduction of the NHSBSP, thereby allowing for the identification of comparison groups with no breast screening activity for the duration of the study. The study found breast cancer mortality was 27% lower adjusted for pretrial rates, in cohort 1 in the two screening centres combined than in the comparison centres (RR: 0.73; 95% CI: 0.63–0.84).

The later study by Johns et al. [9], of 988,090 women aged 49–64 years on 1st January 1991 resident in England and Wales, was unique in design as women moved exposure groups over time, thereby ensuring there was a contemporaneous non-screened comparison group, an issue that has been an obstacle in the design of screening-era cohort studies [5]. Follow-up was, however, limited to a maximum of 15 years. The study estimated that invitation to NHSBSP screening was associated with a 21% reduction in breast cancer mortality (RR: 0.79; 95% CI: 0.73–0.84) after adjustment for age, socioeconomic status and lead-time and breast cancer mortality in first invitation attenders was 32% lower compared with non-attenders after adjustment for age, socioeconomic status and self-selection bias (RR: 0.68: 95% CI: 0.63–0.73) [9].

Aside from the impact of breast screening programmes on reducing breast cancer mortality, an ongoing concern is the extent of overdiagnosis (breast cancer which would not have become clinically apparent in a woman’s lifetime in the absence of screening) [17]. Much of the criticism of previously published estimates of overdiagnosis relates to insufficient follow-up in the post-screening epoch. In the 2012 Independent UK Panel on Breast Cancer Screening Review, the panel stated that the minimum follow-up period needed after screening cessation is 5–10 years [7]. More recently, Chaltiel and Hill have shown that estimates of overdiagnosis from aggregate data tend to be over-inflated, whereas those from studies using individual data on screening exposure tend to be much smaller [18].

To enable assessment of the long-term effectiveness of the NHSBSP on breast cancer mortality and overdiagnosis, the aim of this study was to extend the length of follow-up of the cohort originally studied by Johns et al. [9].

## Methods

### Data sources

The original study cohort collated by Johns et al. [9] consisted of 2,669,328 women aged between 49 and 64 years who were ever registered on one of the NHS databases, known as the Exeter databases, covering 61 local health authorities in England and Wales between 1st January 1988 and 31st December 1994. The study database contained individual level demographic data (age and sex), cancer registration data, death registration data and breast cancer screening histories. Women in the cohort were followed for cancer diagnosis and death until 31st December 2005. Concerns about the completeness of mortality data led to analysis at the time being restricted to a subset of one million women. Detailed information about the data sources have been published previously [9].

As the aim of this current study was to assess the long-term impact of mammography screening on risk of breast cancer incidence and mortality, up-to-date cancer incidence and mortality data were required. NHS number, date of birth and last known postcode (at the time of original data extract in 2008) were submitted to NHS Digital for tracing on the Personal Demographics Service (PDS) database. Cancer and mortality data were then obtained for women successfully traced up to 11th October 2020, with cancer registration obtained by NHS Digital from Public Health England, and mortality registration data from the Office for National Statistics (ONS). Women who had opted out of their data being used for research (through the National Data Opt-Out) were not returned and excluded from the study. No additional screening history data were sought.

### Study design

This study made use of the staggered introduction of the NHSBSP in the UK. The first screening units began inviting women in December 1987, with the roll out continuing until the end of 1994. The earliest date of invitation in the cohort was 10th January 1988. Women aged 50–64 years were invited to attend every three years. The age range has since increased to include women up to the age of 70 years, but this study is limited to women up to and including age 64 years at the time of recruitment.

Women aged 49–64 years entered the study on 1st January 1988, with younger women entering on their 49th birthday until 31st December 1994. All women were uninvited upon entry and remained in the uninvited group until the date of their first invitation, date of breast cancer diagnosis (incidence analysis), date of death or the end of follow-up, 11th October 2020, whichever was earliest. Women only needed to be eligible to receive their first invitation to screening (i.e. aged between 49–64 years) between 1st January 1988 and 31st December 1994, but may not have actually received their first invitation during that period. Women’s first registered breast cancer diagnosis was selected and may have been invasive or ductal carcinoma in situ (DCIS). Given women’s exposure status had the potential to change over time, IBM was used, in which mortality was compared between groups based on their invitation or screening status at the time of diagnosis rather than at the time of death. This means that deaths from breast cancer in the invited group are only included in women diagnosed after their first invitation [5, 19], as screening cannot have an impact on deaths from cancers diagnosed prior to the start of screening [5]. Once in the invited group, women were followed up until date of diagnosis (incidence analysis), date of death or the end of follow-up, whichever was earliest. The same procedure was used for actual exposure to screening, in that rates of death were compared between those with tumours diagnosed before and after first screen respectively.

In the 2017 study by Johns et al. [9], due to incomplete mortality data in the early years of the study (1988–1990) two-thirds of the cohort had to be disregarded. It was hoped that the new iteration of mortality data obtained for this follow-up study would be complete. Dates of death were provided in both the demographics file extracted from the PDS database and the ONS mortality file, with the latter also containing cause of death information. Comparison of the dates of death between the two files revealed that there were in excess of 50,000 women who were listed as having died in the demographics file but did not appear in the mortality file, with the earliest death in the mortality file listed as 4th June 1991. However, over 70% of these deaths were found in the original study database collated by Johns et al. and thus dates of death in the demographics file were taken as correct and used in the final analysis. Johns et al. had undertaken bespoke matching to ascertain causes of death for the early deaths in the cohort as this information was not routinely available from the ONS mortality database. Cause of death was taken from the later mortality file, unless missing, when it was taken from the original study database where available. The dates of death which appeared only in the demographics file were evenly distributed across the study period suggesting no systemic issue with recording of mortality data on the ONS database.

### Statistical analysis

#### Regression modelling

Relative rates assessing the effect of first invitation and first attendance to screening were calculated for both breast cancer incidence and mortality, with overdiagnosis estimated as any residual increase in incidence associated with attendance to screening observed at the end of the follow-up period. Rates were calculated by dividing the number of breast cancer cases or deaths by the number of person-years in each exposure group. Confounding by age and calendar time were adjusted for by fitting a Poisson regression model including age at entry and mean year of follow-up in the uninvited period as continuous covariates. The adjustment for time was based on the uninvited period only as not all women were invited/screened.

The absolute risk reduction was calculated using the method developed by Marmot et al. [7] in which the number needed to be invited (NNI) to prevent one death is given by:

$${NNI}=\,\frac{1}{{R}_{a}(1-{R}_{I})}$$

Where ${R}_{a}$ is the estimated mortality rate in women aged 50 years who are currently expected to die from breast cancer between the ages of 55 and 79 years in the absence of screening, and ${R}_{I}$ is the estimated mortality rate of women invited to screening compared with uninvited women.

Given a participation rate *p*, the number needed to screen to prevent one death is:

$${NNS}={pNNI}$$

${R}_{a}$ was taken to be 2.13 as calculated by Marmot et al. [7].

#### Self-selection bias

Self-selection bias occurs because women who choose not to attend their screening invitation tend to have a higher risk of dying from breast cancer than those who choose to attend, resulting in a bias in favour of screening [20]. This was corrected in the analysis using a modified version of the method proposed by Duffy et al. [21].

The relative rate of breast cancer incidence/mortality for compliers compared with uninvited ψ was estimated as:

$${{\rm{\psi}}} =\frac{P({Diagnosis}\,{or}\,{death}\,{from}\,{breast}\,{cancer}|{Comply}\,{with}\,{screening}\,{invitation})}{P({Diagnosis}\,{or}\,{death}\,{from}\,{breast}\,{cancer}|{Not}\,{invited})}$$

However, ideally an unbiased estimate of the effect of attendance to screening (*R*) was wanted, which is given by the following equation (Duffy et al. [21]):

$$R=\frac{P\,({Diagnosis}\,{or}\,{death}\,{from}\,{breast}\,{cancer}|{Comply}\,{with}\,{screening}\,{invitation})}{P\,({Diagnosis}\,{or}\,{death}\,{from}\,{breast}\,{cancer}|{Not}\,{invited}\,{but}\,{would}\,{attend}\,{if}\,{invited})}$$

With some algebra, it can be shown that:

$$R=\frac{p{{\rm{\psi }}}}{1-(1-p){D}_{r}}$$

where *p* is the proportion complying with an invitation to screening, ${{\rm{\psi }}}$ is the estimated relative rate of breast cancer incidence/mortality for compliers compared with the uninvited and ${D}_{r}$ is the estimated relative rate of breast cancer incidence/mortality for those who were invited but not screened compared with the uninvited (see Supplementary Material). Given the availability of an uninvited comparison group, a population-specific correction factor ${D}_{r}$ was estimated rather than using an estimate from previously published RCTs, as has often been the case in previous studies. The population specific correction factor ${D}_{r}$ was estimated from additional analysis of all-cause mortality excluding breast cancer deaths using non-IBM methodology to enable the inclusion of all deaths in the cohort, not just those in women who had a diagnosis of breast cancer (Table S1). The resulting estimate implies that any change in mortality from causes other than breast cancer in those invited but not screened cannot be due to the screening and therefore, is almost certainly due to self-selection. For the incidence analysis, the outcome of interest for the self-selection factor ${D}_{r}$ was breast cancer incidence. Two self-selection adjusted estimates were calculated, one using an attendance rate *p* of 70%, which is the minimum performance threshold set by the NHSBSP and very close to the average attendance rate achieved by the programme, and one using a cohort-specific estimate for *p*, calculated as the number of person-years accrued in women who attended screening divided by the total number of person-years in all women invited.

Analyses were conducted using Stata version 18 [22] with *p*-values and 95% confidence intervals (CIs) calculated using Poisson regression and a *p*-value of <0.05 considered to indicate statistical significance.

##### Ethical approval

All methods were performed in accordance with the relevant guidelines and regulations, including UK GDPR, the Data Protection Act 2018. Ethical approval has been obtained from NRES Committee London—South East—reference number 02/1/064 and the study received approval under Section 251 of the NHS Act 2006 (to access data without informed consent) through the Confidentiality Advisory Group (CAG)—reference [19]/CAG/0160.

## Results

**Fig. 1:**
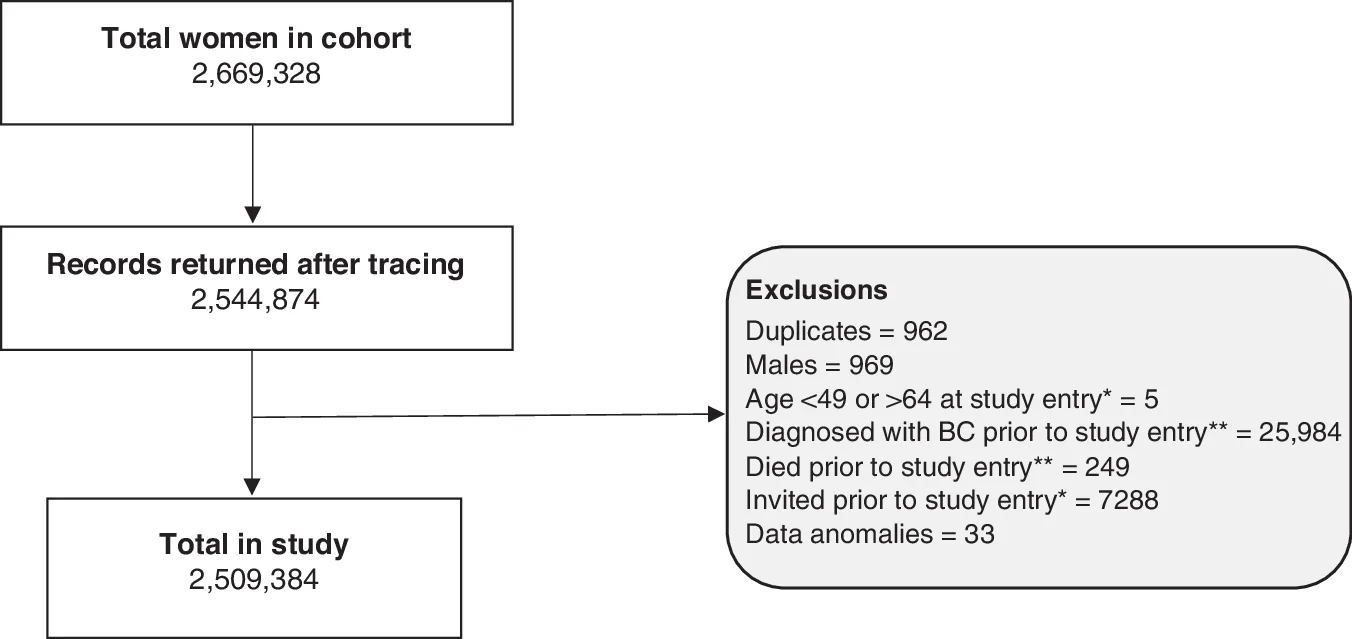
Study flow diagram. *65 or over 1st January 1988 or aged less than 49 31st December 1994. **Study entry is 1st January 1988 for women 49–64 on 01/01/1988 or for women less than 49 on 01/01/1988, it is their 49th birthday (i.e aged between 49 and 64 years between 1st January 1988 and 31st December 1994).

2,544,874 women (95.3%) were successfully traced on the PDS database and demographic data returned (Fig. 1). 962 duplicate records were resolved and a further 34,528 women were excluded from the analysis as they did not meet the study inclusion criteria. Individuals were excluded from the study if they were recorded as male, had a previous diagnosis of breast cancer, died or were invited to screening before 1st January 1988, or were not aged between 49 and 64 years between 1st January 1988 and 31st December 1994. The final analysis dataset provided data on 2,509,384 women (94.0%).

**Fig. 2:**
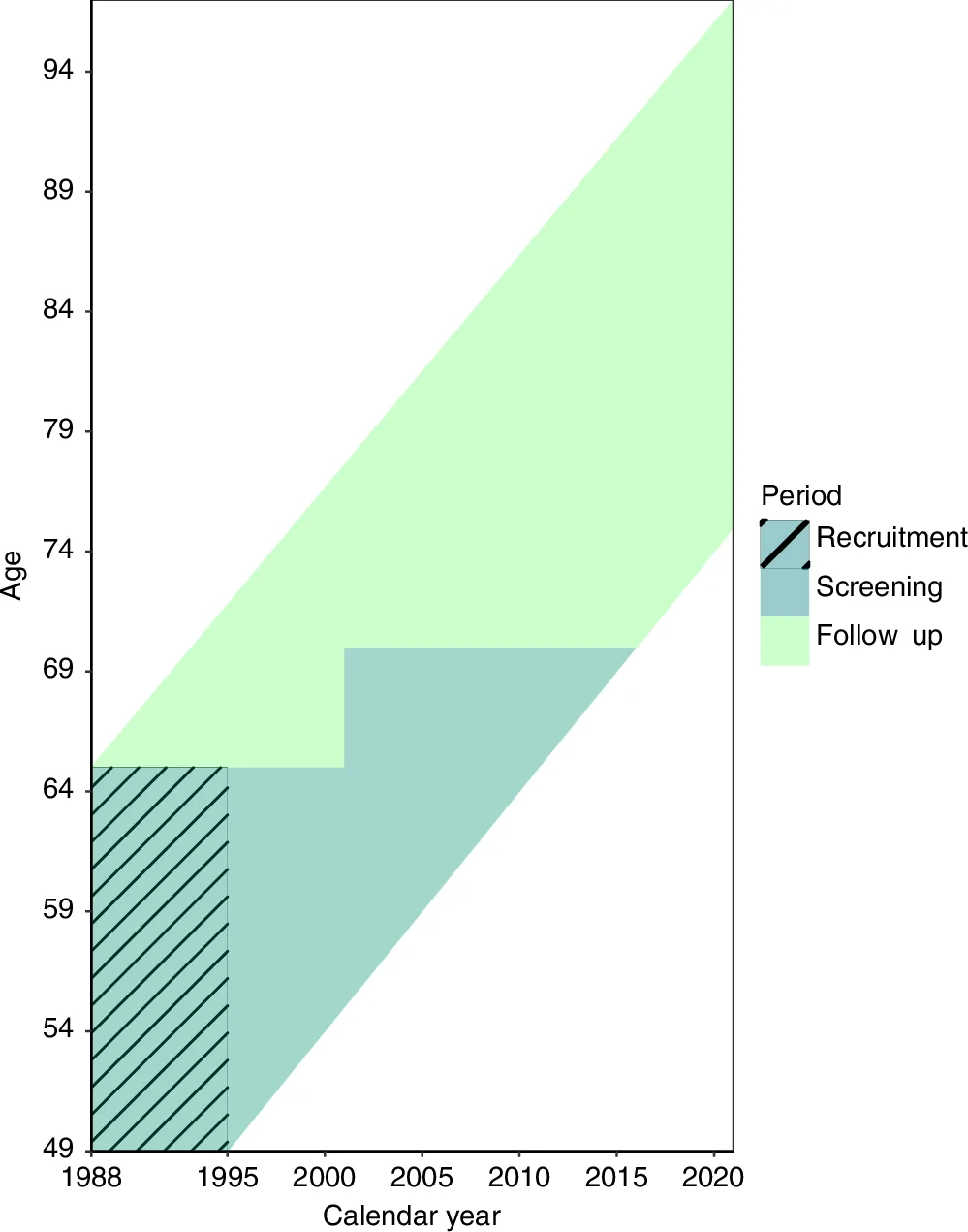
Lexis diagram showing the study population recruitment, screening and follow-up periods.

Table 1 shows characteristics of the final cohort. A total of 1,969,198 (78.5%) women received at least one invitation to breast screening between 1st January 1988 and 25th April 2005. The vast majority (>99%) had received at least one invitation by the end of 1997 (by which time there had been at least one full 36-month screening round in all screening units in England and Wales). The average age at which women entered the study was 53.0 years, with the average age at first invitation of 55.0 years. The average age during the period of observation of uninvited women was 55.5 years and during the period of observation of invited women was 66.8 years. The corresponding average calendar years of observation were 29th October 1990 for uninvited women (midpoint between date of entry and date of invitation or diagnosis/death or end of follow-up) and 29th August 2005 for invited women (midpoint between date of invitation and date of diagnosis/death or end of follow-up). For the majority of the uninvited women, screening had not been introduced in their area before they reached the upper age for invitation (Fig. 2).

**Table 1:** Cohort characteristics

	(*n* = 2,509,384)
Median age at entry, years (IQR; range)	53.0 (49.0–58.8; 49.0–64.9)
Invited ≥1 (%)	1,969,144 (78.5)
Median number of invitations (range)	3 (0–13)
Median age at first invitation, yrs (IQR; range)	55.0 (51.6–59.9; 49.0–78.0)
Screened ≥1 (%)	1,606,462 (64.0)
Median number of screens (range)	3 (0–10)
Median age at first screen, years (IQR; range)	55.4 (52.0–60.0; 49.0–77.9)
Number of breast cancer cases (%)	187,503 (7.5)
Median age at diagnosis, years (IQR; range)	68.4 (61.5–75.6; 49.0–96.1)
Number of breast cancer deaths (%)	46,657 (1.9)
Median age at death, years (IQR; range)	73.7 (66.3–80.5; 49.0–97.6)

### Breast cancer incidence (overdiagnosis)

Table 2 shows the number of women, person-years, breast cancer cases and deaths by screening exposure group. A total of 187,503 first breast cancers were diagnosed in women during the study period: 146,359 in invited women (invited and not yet screened plus women screened, second and third rows of the table) and 41,144 in uninvited women, giving rates of 3.23 and 2.30 per 1000 person-years, respectively and a crude relative rate of 1.41 (95% CI: 1.39–1.42) (Table 3). This relative difference was primarily due to confounding of screening status with age and calendar time (both associated with increasing incidence). Adjustment for confounding by age and time led to a small 6% decrease in breast cancer incidence in women invited to screening compared with uninvited women (RR: 0.94; 95% CI: 0.93–0.95). The crude relative rate was slightly larger when assessing the incidence rates for attendance to screening (RR: 1.48; 95% CI: 1.46–1.49). However, adjustment for confounding by age, time and self-selection bias resulted in a relative rate of 1.04 (95% CI: 1.02–1.07) assuming a participation rate of 70% (which was the average participation rate of women in the NHSBSP during the period of observation), and 1.02 (95% CI: 1.00–1.04) using the study-specific participation rate of 81% respectively. The self-selection correction factor *Dr* calculated from Table 1 was 1.12 (95% CI: 1.10–1.14) (i.e. 22,332/8,673,732 divided by 41,148/17,928,819).

**Table 2:** Number of breast cancer cases and deaths, and corresponding person-years at risk, screening exposure status

Exposure status	Number of women	Breast cancer incidence			Breast cancer mortality		
		Number of person-years	Number of breast cancer cases	Rate of breast cancer cases^a^	Number of person-years	Number of breast cancer deaths	Rate of breast cancer deaths^a^
Uninvited	2,509,384	17,928,027	41,144	2.30	18,373,756	14,516	0.79
Invited	1,969,198	8,674,524^b^	22,336^b^	2.57	8,880,702^b^	8529^b^	0.96
Screened	1,606,462	36,587,664	124,023	3.39	37,932,664	23,612	0.62

^a^Rate per 1000 person-years.

^b^Person-years and outcomes in those invited but not yet screened.

**Table 3:** Crude and adjusted relative risks of invitation and attendance to screening on risk of breast cancer incidence

Exposure Status	Crude relative risk (95% CI)	Adjusted relative risk (95% CI)^a^	Adjusted relative risk (95% CI)^b^	Adjusted relative risk (95% CI)^c^
Invitation to screening	1.41 (1.39–1.42)	0.94 (0.93–0.95)	NA	NA
Attendance to screening	1.48 (1.46–1.49)	0.99 (0.97–1.00)	1.04 (1.02–1.07)	1.02 (1.00–1.04)

*NA* not applicable.

^a^Adjusted for age at study entry and mean year of follow-up in the uninvited group.

^b^Adjusted for age at study entry and mean year of follow-up in the uninvited group and self-selection bias using *p* = 0.70 and *Dr *= 1.12 (95% CI: 1.10–1.15).

^c^Adjusted for age at study entry and mean year of follow-up in the uninvited group and self-selection bias using *p* = 0.81 and *Dr *= 1.12 (95% CI: 1.10–1.15).

### Breast cancer mortality

Of the 187,503 women who were diagnosed with breast cancer, 46,657 died of the disease; 32,141 in women invited for screening and 14,516 in women who received no invitation prior to diagnosis. This led to rates of 0.69 and 0.79 per 1000 person-years, respectively and thus women invited to screening had a crude 13% lower risk of dying from breast cancer when compared to uninvited women (RR: 0.87; 95% CI: 0.85–0.89) (Table 4). This increased to a 28% reduction in mortality after adjustment for age and time (RR: 0.72; 95% CI: 0.71–0.74). For women who attended at least one screen, the risk reduction was higher than for invitation, with a 21% reduction in breast cancer mortality compared with those never invited (RR: 0.79, 95% CI: 0.77–0.80). This increased after adjustment for age, time and self-selection bias, with a 32% reduction when assuming the average national programme participation rate of 70% (RR: 0.68; 95% CI: 0.71–0.77) and estimating the self-selection factor *Dr* as 1.05 (95% CI: 1.04–1.06) (Table S1). Assuming a study-specific participation rate of 81%, the estimated reduction in breast cancer mortality was 33% (OR: 0.67; 95% CI: 0.65–0.69).

**Table 4:** Crude and adjusted relative risks of invitation and attendance to screening on risk of breast cancer mortality

Exposure status	Crude relative risk (95% CI)	Adjusted relative risk (95% CI)^a^	Adjusted relative risk (95% CI)^a^	Adjusted relative risk (95% CI)^a^
Invitation to screening	0.87 (0.85–0.89)	0.72 (0.71–0.74)	NA	NA
Attendance to screening	0.79 (0.77–0.80)	0.67 (0.65–0.68)	0.68 (0.66–0.71)^b^	0.67 (0.65–0.69)^c^

*NA* not applicable.

^a^Adjusted for age at study entry and mean year of follow-up in the uninvited group.

^b^Additionally adjusted for self-selection bias assuming a participation rate of 70% and *Dr *= 1.05 (95% CI: 1.04–1.06).

^c^Additionally adjusted for self-selection bias assuming a participation rate of 81% and *Dr *= 1.05 (95% CI: 1.04–1.06).

The absolute benefit of invitation to and attendance at screening was estimated using the method of Marmot et al. [7]. Based on data in Table 3, the estimated adjusted mortality reduction associated with invitation to screening was 28%. Given an overall estimated breast cancer mortality rate in women of screening age of 2.13 [7], the number needed to be invited aged 50 – 70 years to prevent one breast cancer death would be 168 women. The number needed to screen to prevent one death from breast cancer, assuming an attendance rate of 70%, would therefore be 118 women and assuming a population-specific attendance rate of 81%, would require 136 women to be screened.

## Discussion

In this study, the long-term impact of the NHSBSP on breast cancer incidence and breast cancer mortality was estimated using a cohort of over two and a half million women followed up for more than 30 years [9].

There was a 28% reduction in breast cancer mortality in women invited to screening compared to those who were never invited, after adjustment for age and time. Actually being screened was associated with a larger 33% reduction in mortality from breast cancer. After adjustment for age, calendar period and potential self-selection bias, there was a 4% excess of breast cancer diagnoses associated with being screened (this includes DCIS). Women were followed for a maximum of 32.8 years making this one of the longest prospective population-based studies to be conducted to date and included over 5-years of follow-up after the cessation of screening for even the youngest women in the study. The long-term effect of both invitation and attendance to screening were assessed in relation to breast cancer incidence, thus providing an estimate of overdiagnosis, and mortality.

The 28% reduction in mortality associated with invitation is larger than that originally reported by Johns et al. [9] based on 15-years follow-up, but is in keeping with results from the Swedish Two County Trial based on 29-years of follow-up [3], as well as other IBM studies with contemporaneous comparison groups [23, 24]. Breast cancer mortality was 33% lower in women who attended at least one screen compared to those never invited, which is comparable to the 32% reduction reported by Johns et al. While this estimate is more conservative than the 38% reported by Maroni et al. [12], their study of the NHSBSP had a case-control design and as such, may have overestimated effectiveness [25]. Given the availability of mortality data for other causes, *Dr* was calculated using non-IBM analysis of all deaths excluding those from breast cancer. The rationale for using non-breast cancer mortality as the outcome is that any additional risk of death observed in the invited but not screened group cannot be related to the intervention of breast cancer screening, and is unlikely to be due to a behavioural effect of invitation given that breast cancer deaths are excluded, and therefore must be due to self-selection. *Dr* was estimated to be 1.05 when using all-cause mortality excluding breast cancers as the endpoint, and this led to an adjusted estimated effect of attendance at screening of 33%, which is in line with other published estimates [4].

The estimated numbers needed to be invited and screened over a 20-year period to prevent one death from breast cancer (168 and 118–136, respectively) were generally smaller than those estimated by Marmot et al. in their independent review (235 and 180, respectively) [7]. The latter may be conservative due to the inclusion of the Canadian Trials in which subversion of randomisation has been reported [26]. The estimated number needed to screen was significantly lower than the estimate by Johns et al. in their previous analysis of the same cohort [9], which is to be expected with the extended follow-up and larger absolute numbers of deaths prevented. However, they were more conservative than the 111 estimated by Maroni et al. in their UK case-control study [12].

Comparison of incidence rates in screened women with those never invited, adjusted for age, time, and self-selection bias, suggests the rate of overdiagnosis associated with the programme to be between 2 and 4%, and provides arguably the most reliable estimate that can be derived from the programme at the moment. This is smaller than the estimate in the review by Marmot et al. [7], probably because of the longer follow-up in this cohort study. Our estimate is slightly larger than the <1% reported by Johns et al. [9] but consistent with the 3.7% estimated in a recently published case-control study by Blyuss et al. [27]. It should be noted, however, that the estimate derived here is based on screening activity carried out in the early years of the programme, when one-view film mammography was in use, and women were only screened until the age of 64 years. Current screening protocol involves the use of two-view digital mammography, which is more sensitive than the previous protocol, thus increasing cancer detection rates, and women are offered screening up to the age of 70 years. Therefore, overdiagnosis associated with the current programme may be slightly more than observed here. However, the estimate is modest and is consistent with Chaltiel and Hill’s observation that small estimates of overdiagnosis are observed when individual data on screening exposure are available [18].

The design of the study was such that women’s exposure status had the potential to change over time. This was advantageous in respect of minimising the potential for bias (particularly lead time bias) across exposure groups which has been an issue in other IBM studies [5]. However, given that this study spanned over three decades, with women’s ages ranging from 49.0 years at entry up to 97.8 years by the end of follow-up in 2020, during which time there have been improvements in diagnostic and treatment techniques [28] as well as cancer survival [29], it was inevitable that the results would be confounded by age and time. This was addressed by fitting a Poisson regression model with mean year of follow-up in the uninvited women and age at entry as covariates in the model however, there may be additional confounders that we have been unable to adjust for due to limited data availability. Also, age and time confounding may be more complicated than a simple log-linear regression relationship with the outcome.

The latest date of invitation was 25th April 2005. Reasons why women may not have received their first invitation until the 2000s include prior screening outside of England and Wales, prior withdrawal from screening due to ill health, not registered with the NHS in the 1990s (e.g. due to emigration), however numbers were minimal. Some women remained uninvited throughout the study period. The introduction of screening was staggered over a 7-year period with different regions starting early and late. For the majority of the uninvited women, screening had not been introduced in their area before they reached the upper age for invitation (Fig. 2).

The main limitation of this study was the possibility of incomplete mortality data. The new mortality data extract from the ONS was obviously incomplete as it contained no deaths prior to June 1991. However, this was augmented by the PDS data, and there was a high level of matching between dates of death in the original cohort data extract from 2008 and the recent extract from the PDS database, confirming the validity of the original data. In addition, the new data extract contained hundreds of thousands of deaths from 2008 onwards of previously excluded women who entered the study in between 1988 and 1990, indicating that, by definition, those women were alive in the early years of the study, and their mortality data were not indeed missing. Nevertheless, there was still the possibility of some incomplete mortality data from the early years of the study, and thus the results presented here should be interpreted bearing this in mind. Missing mortality data would have most impact on the uninvited group given that the missing data pertains to the early years of follow-up. However, as this exposure group is large, it would have a minimal impact on the overall rates. A small number of these missing deaths could be breast cancer deaths. This might mean a slight underestimate of the reduction in breast cancer mortality with invitation or screening, since rate of missing deaths would be differentially higher in the uninvited population.

Additionally, 8.8% of deaths with a cause of death listed as breast cancer had no corresponding cancer registration record. It is probable that, in at least some of these instances, the women were diagnosed at death. The distribution of dates of death for these subjects were skewed, with a higher number occurring in the earliest years of the study, perhaps reflecting the fact that the recording practices of cancer registration data were poorer in earlier years and thus records were more likely to be incomplete. These women were kept in the study until date of death.

In conclusion, the over-riding strength of this study is its use of individual-level exposure and outcome data on more than 2.5 million women to estimate the long-term impact of the NHSBSP on breast cancer incidence and breast cancer mortality. Members of the cohort were followed up for more than 30 years, making it one of the largest and longest studies of its kind. It provides evidence of the continued beneficial effect of mammography screening persisting for many years and suggests that the amount of overdiagnosis associated with screening is small.

## Supplementary information


Supplementary Material


## Data Availability

The data used in this study are subject to data sharing agreements with NHS England (formerly NHS Digital) and were accessed via the Data Access Request Service (DARS) under project reference NIC-147747-KRTQ8-v5.1. The data are not publicly available due to data protection and confidentiality requirements. Researchers wishing to access similar data can apply through the NHS England DARS process: https://digital.nhs.uk/services/data-access-request-service-dars.
