# Supplementary material for: Long-term effectiveness and overdiagnosis in the NHS breast screening programme: a cohort study with up to 33 years of follow-up
Source: Br J Cancer. 2026 Jun 26;135(6):964–71. doi: 10.1038/s41416-026-03493-z (PMC13534734; doi:10.1038/s41416-026-03493-z)
Supplement: Supplementary file 1 [file 41416_2026_3493_MOESM1_ESM.docx]

# Supplementary Material

Self-selection bias is an important issue in screening studies as evidence has shown that women who choose not to attend their screening invitation have poorer health and breast cancer survival outcomes than those who choose to attend.

Duffy et al. proposed a self-selection adjustment based on the comparison of attenders at screening with non-attenders (21). However, the effect of attendance in this study was measured by comparing attenders at screening with an uninvited population. Therefore, using the same methodology as Duffy et al., a modified self-selection adjustment was calculated.

The effect of attendance at screening adjusted for self-selection can be written as:

$$R=\frac{P(Diagnosis or death from breast cancer|Comply with screening invitation)}{P(Diagnosis or death from breast cancer|Not invited but would attend if invited)}$$

Let event $D$ be the outcome (incidence of or death from breast cancer), $S$ denote screened, and $NI$ not yet invited.

From the data, $R^{*}$ can be directly calculated, where:

$$R^{*}=\frac{P(D|S)}{P(D|NI)}$$

However, $R$ is required, where:

$$R=\frac{P(D|S)}{P(D|NIW)}$$

where NIW denotes not invited but would have attended if invited. Now,

$$P\left( D | NI \right)=pP\left( D | NIW \right)+\left( 1-p \right)P(D|NS)$$

where p is the proportion complying with an invitation to screening and $NS$ denotes never screened. Thus,

$$P\left( D | NIW \right)=\frac{P\left( D | NI \right)-\left( 1-p \right)P(D|NS)}{p}$$

So:

$$R=\frac{pP(D|S)}{P\left( D | NI \right)-\left( 1-p \right)P(D|NS)}$$

$$R^{-1}=\frac{P(D|NI)}{pP(D|S)}-\frac{\left( 1-p \right)P(D|NS)}{pP(D|S)}$$

$$=\frac{1}{pR^{*}}-\frac{\left( 1-p \right)P\left( D | NS \right)P\left( D | NI \right)}{pP\left( D | S \right)P\left( D | NI \right)}$$

$$=\frac{1}{pR^{*}}-\frac{(1-p)D_{r}}{pR^{*}}$$

Where:

$$D_{r}=\frac{P(D|NS)}{P(D|NI)}$$

Therefore,

$$R=\frac{pR^{*}}{1-(1-p)D_{r}}$$

$D_{r}$ was calculated using non-IBM methodology and with all-cause mortality excluding breast cancers as the endpoint of interest. $D_{r}$ was calculated 191,606/8,926,144 divided by 373,048/18,211,037, that is 1.05 (95% CI: 1.04 – 1.06) (Table S1).

**Table S1:**

|  | Number of women | Number of person-years | Cause of death |
| --- | --- | --- | --- |
|  |  |  | All-causes excluding breast cancer |
| Uninvited | | | |
| Number | 2,509,384 | 18,211,037 | 373,048 |
| Rate^a^ | – | – | 20.5 |
| Invited but not screened | | | |
| Number | 1,978,564 | 8,926,144 | 191,606 |
| Rate^a^ | – | – | 21.5 |

^a^ Rate per 1,000 person-years

**Figure S1 (a-c): Accrual of person-years and breast cancer cases in different exposure groups**

All women were uninvited upon entry and remained in the uninvited group until the date of their first invitation, date of breast cancer diagnosis, date of death or the end of follow-up, 11th October 2020, whichever was earliest (figure a). Once in the invited group, women were followed until date of diagnosis (incidence analysis), date of death or the end of follow-up, whichever was earliest (figure b). The same procedure was used for actual exposure to screening (figure c). If diagnosed, women were followed for date of breast cancer death (mortality analysis), date of death or the end of follow-up (dashed line).

a. If a woman was diagnosed whilst uninvited, X person-years were accrued in the uninvited group.

b. If diagnosed whilst invited, X person-years were accrued in the uninvited group and Y person-years were accrued in the invited group.

c. If diagnosed after their first screening attendance, X person-years were accrued in the uninvited group, Y person-years were accrued in the invited group and Z person-years were accrued in the screened group.
